# Mean global field power is reduced in infantile epileptic spasms syndrome after response to vigabatrin

**DOI:** 10.3389/fneur.2024.1476476

**Published:** 2024-10-25

**Authors:** Arjun Nair, Joycelyne Ewusie, Rowan Pentz, Robyn Whitney, Kevin Jones

**Affiliations:** ^1^The Division of Neurology, Department of Pediatrics, McMaster Children’s Hospital, Hamilton, ON, Canada; ^2^The Department of Health Research Methods, Evidence and Impact McMaster University Hamilton, Hamilton, ON, Canada

**Keywords:** computational EEG biomarkers, functional connectivity, mean global field power, vigabatrin, infantile epileptic spasms syndrome

## Abstract

**Purpose:**

Infantile epileptic spasms syndrome (IESS) is associated with abnormal neuronal networks during a critical period of synaptogenesis and brain plasticity. Hypsarrhythmia is a visual EEG biomarker used to diagnose IESS, assess response to treatment, and monitor relapse. Computational EEG biomarkers hold promise in providing unbiased, reliable, and objective criteria for clinical management. We hypothesized that computational and visual EEG biomarkers of IESS would correlate after treatment with vigabatrin and that these responses might differ between responders and non-responders.

**Methods:**

A retrospective analysis was conducted at a single center, involving children with IESS at initial diagnosis and following first-line treatment with vigabatrin. Visual EEG biomarkers of hypsarrhythmia were compared with computational EEG biomarkers, including spike and spike fast-oscillation source coherence, spectral power, and mean global field power, using retrospective analysis of EEG recorded at initial diagnosis and after vigabatrin treatment. Responders and non-responders were compared based on the characteristics of their follow-up EEGs.

**Results:**

In this pilot study, we observed a reduction in the EEG biomarker of hypsarrhythmia/modified hypsarrhythmia from 20/20 (100%) cases at the initial diagnosis to 9/20 (45%) cases after treatment with vigabatrin, indicating a 55% (11/20) responder rate. No significant difference in spike frequency was observed after treatment (*p* = 0.104). We observed no significant differences after treatment with vigabatrin in the computational EEG biomarkers that we assessed, including spike source coherence at 90% (*p* = 0.983), spike source coherence lag range (*p* > 0.999), spike gamma source coherence at 90% (*p* = 0.177), spike gamma source coherence lag range (*p* > 0.999), spectral power (0.642), or mean global field power (0.932). However, when follow-up EEGs were compared, there was a significant difference in mean global field power (*p* = 0.038) between vigabatrin responders and non-responders. In contrast, no such difference was observed for spike source coherence at 90% (*p* = 0.285), spike course coherence lag range (*p* = 0.819), spike gamma source coherence at 90% (*p* = 0.205), spike gamma source coherence lag range (*p* > 0.999), or spectral power (*p* = 0.445). Finally, our treated group did not differ significantly from healthy controls at initial diagnosis or follow-up in terms of spectral power (*p* = 0.420) or mean global field power (0.127).

**Conclusion:**

In this pilot study, we show that mean global field power is a computational EEG biomarker that is significantly reduced in IESS after treatment with vigabatrin. Although computational EEG biomarkers of network connectivity using spike source coherence appear to be a promising tool, future studies should further explore their potential for assessing treatment responses in IESS.

## Highlights

- In this pilot study, we demonstrate that mean global field power is a computational EEG biomarker that is significantly reduced in infantile epileptic spasms syndrome (IESS) after treatment with vigabatrin.

## Introduction

Infantile epileptic spasms syndrome (IESS) is a rare developmental and epileptic encephalopathy (DEE) of infancy, clinically characterized by epileptic spasms and an interictal electroencephalography (EEG) pattern known as hypsarrhythmia, which is often accompanied by developmental delay (1). The onset of IESS typically occurs between 3 and 12 months of age, with a range extending from 1 to 24 months. The pathophysiology of IESS is poorly understood. During infancy, neuronal glutaminergic excitation predominates, promoting synaptogenesis in cortical and limbic neuronal networks, with increased synaptic and spine density, as well as increased excitatory ion channels and transporter activity. At the same time, GABAergic inhibition is reduced during early life. This heightened hyperexcitability increases the risk for seizures and epileptogenesis during this critical period of brain development (2, 3). One hypothesis suggests that IESS results from temporal desynchronization of developmental processes, leading to disturbed brain function (4). This disruption may be due to structural, metabolic, genetic, infectious, immune, or unknown causative factors (1, 5).

At our institution, high-dose vigabatrin is the preferred first-line agent for the treatment of IESS because of its better short-term side effect profile and relative ease of use (6). Vigabatrin is a selective, enzyme-activated, irreversible inhibitor of gamma-aminobutyric acid transaminase. Increased availability of gamma-aminobutyric acid within the synaptic cleft increases the efficacy of inhibitory interneurons (7). The response rates of vigabatrin in the first line treatment of IESS not related to tuberous sclerosis complex range between 9 and 50%, which is lower than with hormonal therapy (6, 8).

Long-term follow-up studies have shown that three-quarters of children with IESS will have an unfavorable outcome, and two-thirds will continue to have seizures (8). The outcome is predicted primarily by the underlying etiology, which leads to a DEE. Timely resolution of the epileptic encephalopathy is a modifiable predictor of outcome. This requires early recognition of epileptic spasms, accurate diagnosis of hypsarrhythmia, treatment with hormonal therapy or vigabatrin, and clinical response to treatment with resolution of hypsarrhythmia (8).

Visual EEG biomarkers, based on the human identification of characteristic EEG patterns, are used in diagnosing IESS, evaluating treatment responses, and monitoring for relapse (9). Hypsarrhythmia is a visual EEG biomarker defined by randomness, high voltage, disorganization, and multifocal independent epileptiform discharges (spikes and sharp waves) (10, 11). Variations of the hypsarrhythmia pattern have been observed, referred to as modified hypsarrhythmia. Hussain et al. (12–14) reported poor interrater reliability in assessing hysparrhythmia. Consequently, there continues to be a need for developing unbiased, reliable, objective criteria to determine the presence of hypsarrhythmia or its modified forms to guide clinical management decisions for patients with IESS.

Computational EEG biomarkers, which use computers to calculate quantitative EEG features, are emerging as a promising research area to address this need (9). Spectral power is a measure of EEG amplitude in distinct frequency bands derived from a Fourier transform. Studies have indicated that children with IESS exhibit significantly higher interictal EEG amplitudes and spectral power across standard frequency bands than healthy controls while awake and asleep (15, 16).

Functional brain connectivity networks can be estimated using cross-correlation, which assesses the correlation between two EEG signals shifted in time relative to each other. Significant correlations (excluding zero time lag, which represents volume conduction) may be measured up to 200 ms and are generally considered to indicate transsynaptic neuronal activity (9). Coherence is another frequency-specific measurement of functional connectivity.

Functional connectivity networks are stronger in IESS than controls, and treatment responders have reduced functional connectivity compared to non-responders. Hypsarrhythmia has been shown to be associated with an increased long-electrode distance, sensor coherence during sleep. EEG electrode sensor coherence measures the degree of functional (cortical and subcortical) network connectivity between regions (15). Functional network connectivity measured by EEG coherence has been identified as a potential quantitative marker of electrographic treatment response in comparison to visual EEG analysis (17). Shrey et al. (17) found that subjects with epileptic spasms had stronger functional networks than controls. Clinical treatment responders demonstrated reduced functional connectivity, while non-responders demonstrated increased or minimally decreased functional connectivity. Interictal discharges in IESS subjects are associated with a global increase in connection strength without altering the functional connectivity network structure (18). In children with IESS secondary to perinatal arterial ischemic stroke, strong connectivity between intra- and interhemispheres generates hypsarrhythmia through epileptogenic cortical-subcortical and transcallosal networks (19).

Scalp EEG fast oscillations in the gamma frequency band (40–80 Hz) occur more frequently and with higher energy during sleep in children with IESS than in healthy controls (20, 21). In children with tuberous sclerosis complex, fast oscillations associated with interictal spikes increased at the onset of IESS and decreased after steroid treatment; however, fast oscillations not associated with spikes did not change (22).

The integration of computational and visual EEG biomarkers by epileptologists in clinical epilepsy practice remains challenging. Commercial software packages using proprietary algorithms, such as Curry by Compumedics Neuroscan, are available; however, they require specialized computational EEG expertise to configure and have not yet been widely adopted in clinical settings (9). In this study, we aimed to compare computational EEG biomarkers with visual EEG biomarkers for both the initial diagnosis and treatment response to vigabatrin in children with IESS, calculated using Curry Compumedics Neuroscan software. We hypothesized that quantitative EEG biomarkers (including spectral power, mean global field power, interictal spike, and spike fast oscillation source connectivity) and visual EEG biomarkers (such as hypsarrhythmia and modified hypsarrhythmia) would change with vigabatrin treatment and differ between responders and non-responders.

## Materials and methods

A single-center retrospective health record analysis of children with newly diagnosed infantile spasms was conducted at McMaster Children’s Hospital between January 2002—and July 2015.

### Inclusion criteria

The inclusion criteria for the study were as follows: a clinical diagnosis of epileptic spasms in children between the ages of 2–24 months, presence of infantile spasms and hypsarrhythmia or modified hypsarrhythmia on initial EEG, first-line treatment of infantile spasms with high dose vigabatrin, a follow-up after approximately 14 days from initiation of treatment with repeat EEG, and a follow-up period of at least 6 months to a maximum of 3 years. The control group consisted of children between 2 months and 2 years, with no neurological diagnosis and one normal EEG.

### Patient data

The following variables were analyzed: sex, gestational age, age at onset of infantile spasms, etiology of infantile spasms, treatment medication, treatment lag time, clinical response to first-line treatment, electrographic response to first-line treatment, follow-up duration, infantile spasms relapse, seizure resolution at the end of follow up.

### EEG selection

EEGs obtained at the time of diagnosis and at follow-up after approximately 14 days from initiation of treatment during sleep were included. Control EEGs consisted of one routine EEG while asleep.

### EEG analysis

EEG records were de-identified and reviewed by a Canadian Society of Clinical Neurophysiologists (CSCN)-certified electroencephalographer (KJ) blinded to etiology. EEG data were reported as hypsarrhythmia or modified hypsarrhythmia, slow background, multiple independent spike foci (MISF), or normal, and it was compared to the retrospective EEG reports for consensus. In cases where there was a difference of opinion between the initial report and the reviewer about the presence or absence of hypsarrhythmia/modified hypsarrhythmia, a Burden of AmplitudeS and Epileptiform Discharges (BASED) score of >3 was used for confirmation (14).

### EEG source coherence analysis

**Figure 1:**
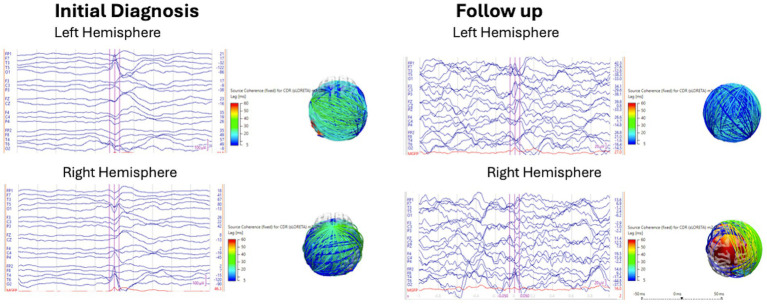
Spike source coherence 1-30 Hz at initial diagnosis and follow up.

This EEG source coherence analysis was performed using Curry 9 software, provided by Compumedics Neuroscan. Manual spike marking of interictal discharges from the left hemisphere (spike 1) and right hemisphere (spike 2) was performed by a CSCN-certified electroencephalographer (KJ) on EEG at initial diagnosis and at follow-up with a 1–30 Hz filter. Marked spikes were reviewed with field maps to confirm hemispheric localization, and discordant spikes were removed. Spike averaging from each hemisphere was performed with noise estimation of −1,000 ms to −100 ms. A realistic, standardized pediatric magnetic resonance imaging (MRI) head model was generated using the boundary element method. Spike source coherence was generated for the averaged spikes from each hemisphere using a source location 3-dimensional (3-D) grid with grid spacing of 15 mm, and a fixed standardized low-resolution electromagnetic tomography (sLORETA) current density map clipped below 90%, with zero lag removal switched on (to exclude volume conduction), with a source minimum lag time range of 5 ms and a maximum lag time range of 60 ms. Source coherence connectivity maps and lag times depicted on a 3-dimensional head model were reviewed for the presence or absence of source connectivity clipped below 90% and lag time ranges of 5–20 ms, 5–40 ms, and 40–60 ms at initial diagnosis and follow-up (Figure 1).

**Figure 2:**
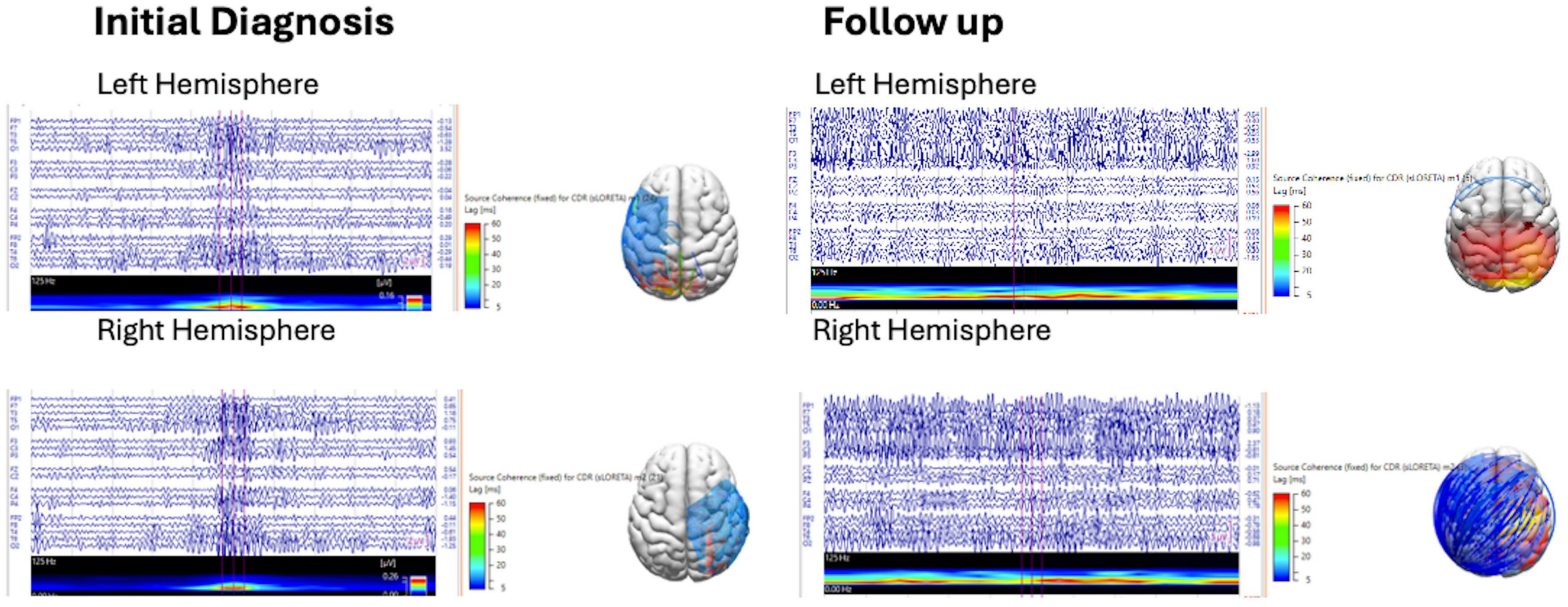
Spike gamma source coherence 30-70 Hz at initial diagnosis and follow up.

The marked spikes were then reanalyzed by applying a gamma filter between 30 and 70 Hz, and spike averaging was performed for each hemisphere. A 200 ms epoch at the averaged spikes was delineated, and a short-time fast Fourier transform (SFTT) analysis was applied to ensure that there was a gamma activity during the marked epoch. Interictal spike gamma source coherence was generated based on the interictal spike source coherence (Figure 2).

### Spectral power and mean global field power analysis

**Figure 3:**
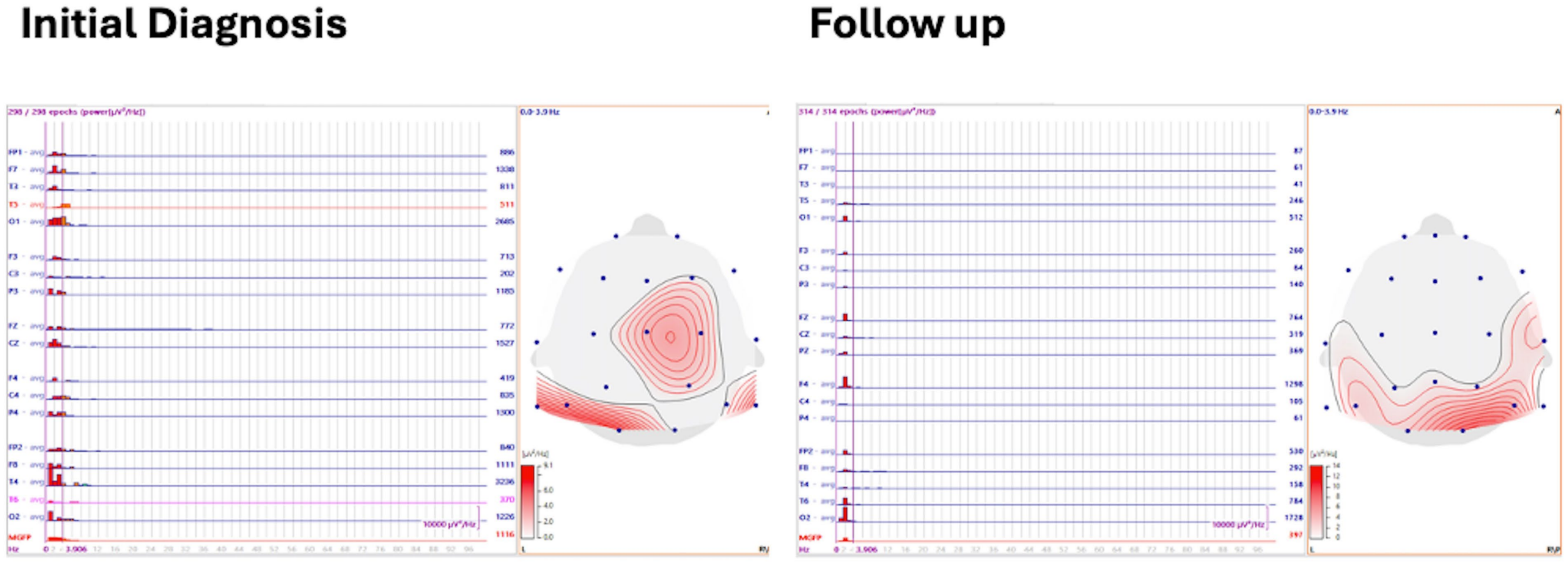
Mean global field power and spectral power at initial diagnosis and follow up.

Three to five minutes of asleep EEG was analyzed with 1 s back-to-back epochs averaged with spectral power frequency, and the delta frequency band was defined and displayed on a 2-D topographical map. Mean global field power (MGFP) was also recorded (Figure 3).

Control EEG amplitudes were assessed using spectral power and MGFP. Spike source coherence and spike gamma source coherence were not evaluated, as the control EEGs had no interictal spikes.

### Objectives

The study aimed to assess the following objectives:

1. The change in EEG biomarkers after treatment (initial vs. follow-up).
2. The difference between vigabatrin EEG responders and non-responders. (The analysis was also performed and adjusted for sex and age.)
3. The change in spectral power and MGFP between the control group and the IESS group at baseline and follow-up.

### Outcome measures

The outcome measures are as follows:

1. Complete cessation of epileptic spasms.
2. Resolution of hypsarrhythmia/modified hypsarrhythmia.
3. Correlation between interictal spike and spike gamma source coherence and lag time range, spectral power, and MGFP and EEG at initial diagnosis and follow-up after treatment with vigabatrin.

### Definition of terms

Clinical response: This is defined as the cessation of epileptic spasms, no reported epileptic spasms for at least 48 h, including the period up to day 14 post initiation of treatment.

EEG response: This refers to the total resolution of hypsarrhythmia or modified hypsarrhythmia pattern observed during follow-up, EEG at approximately day 14.

### Statistics

Categorical variables were assessed using the chi-squared test and Fisher’s exact test. Continuous variables were assessed using independent student’s *t*-test reported at an estimate of the 95% confidence intervals. EEG biomarkers were summarized as count (%) for binary measures and median (Q1, Q3) for continuous measures.

### Objective 1

Change in EEG biomarkers from initial diagnosis was assessed using a logistic mixed effects model for the binary outcomes, and a quantile regression model was used to assess continuous outcomes. The change effect was expressed as the odds ratio of 95% confidence interval (95% CI) or the difference in medians (95% CI), respectively. A mixed effects model was used to account for the repeated measures. All models were adjusted for baseline/initial diagnosis.

### Objective 2

The difference in follow-up vigabatrin EEG between responders and non-responders was assessed using Fisher’s exact test and the Wilcoxon–Mann–Whitney *U* test for binary and continuous outcomes, respectively. Effect estimates were again summarized as odds ratio (OR) (95% CI) or difference in proportion (95% CI) for binary outcomes and difference in location (95% CI) for continuous outcomes.

### Objective 3

To investigate the differences in spectral power and MGFP between the control group and the intervention group at the initial diagnosis and follow-up, we conducted a Kruskal–Wallis analysis since our data did not meet the assumptions required for analysis of variance to perform analysis of variance (ANOVA). Post-hoc analyses were planned for any significant results.

For all analyses, a *p*-value of 0.05 was deemed statistically significant.

## Results

### Patient data

A total of 20 subjects were included in the study. The clinical data are presented in Table 1. Moreover, 11 subjects (55%) responded to vigabatrin by visual EEG analysis. There was no statistical difference between vigabatrin responders and non-responders with regard to sex, gestational age, age at diagnosis, treatment lag time, etiology follow-up duration or final follow-up age, epileptic spasms relapse, and seizure resolution at the end of follow-up.

**Table 1:** Patient data.

Variable	Entire cohort 20	VBG responders (%) 11 (55)	VGB non responders (%) 9 (45)	*p*-value
Sex, *N* (%)				
Female	11 (55)	7 (64)	4 (44)	0.65
Male	9 (45)	4 (36)	5 (56)	
Gestational age, months median (IQR)	39.5 (38–40)	39.5 (38–40)	39.5 (38–41)	0.52
Age at diagnosis, months median (IQR)	6 (4.25–9)	6 (3–6.5)	9 (5.5–9.75)	0.11
Treatment lag time, months median (IQR)	3.5 (1.6–6)	5 (2–6)	3 (1–5.5)	0.45
Etiology, *N* (%)				
Structural	6 (30)	4 (36)	2 (22)	0.448
Metabolic	0			
Genetic	2 (10)		2 (22)	
Immune	0			
Symptomatic	8 (40)	4 (36)	4 (44)	
Unknown	12 (60)	7 (64)	5 (55)	
Follow-up (months), median (IQR)	15.5 (12–20)	14 (12–20)	16 (16–23)	0.3
Final follow up age, months median (IQR)	21.5 (18–28)	20 (16–26)	23 (19–30.5)	0.76
Relapse, *N* (%)	10 (50)	6 (55)	4 (44)	1
Seizure resolution at end of follow-up *N* (%)	9 (45)	5 (45)	4 (44)	1

### EEG biomarkers at initial diagnosis

At initial diagnosis, the visual biomarker of hypsarrhythmia was present in 20/20 (100%) cases, and spikes were present in 20/20 (100%) cases. Spike source coherence, at 90%, was present in 20/20 (100%) of cases with a median lag range of 20 ms (IQR 20–40 ms). Spike gamma source coherence at 90% was present in 20/20 (100%) cases with spike gamma source coherence lag range of 20 ms (IQR 20, 20 ms). Maximum spectral power was represented as a median of 12 μV^2^/Hz (IQR 8.4, 15.5 μV^2^/Hz), and mean global field power was found to be 546 μV^2^ (IQR 373.75, 886.25 μV^2^).

### EEG biomarkers after treatment with vigabatrin

There was a significant reduction in the visual EEG biomarker of hypsarrhythmia/modified hypsarrhythmia from 20/20 (100%) cases at the initial diagnosis to 9/20 (45%) cases after treatment with vigabatrin.

The spike source coherence at 90% network size was increased in 5/17 (29%) of cases, unchanged in 8/17 (47%) cases, and decreased in 4/17 (24%) cases after treatment. The spike in gamma source coherence at 90% network size was increased in 11/15 (73%) cases, unchanged in 2/15 (13%), and decreased in 2/15 (13%) after treatment. The EEG biomarkers after treatment with vigabatrin are presented in Table 2.

**Table 2:** Change in EEG biomarkers after treatment with vigabatrin.

EEG biomarker	Initial diagnosis (*n* = 20)	Follow-up (*n* = 20)	Change from initial diagnosis	*p*-value
Visual EEG				
Hypsarrhythmia; *n* (%)	20 (100)	9 (45)		
Spikes; *n* (%)	20 (100)	17 (85)	0.12 (0.001, 1.43)	0.104
Computational EEG				
Spike source coherence @ 90%; *n* (%)	20 (100)	17 (85)	0.85 (0.005, >99.9)	0.943
Missing		3		
Spike source coherence lag range; median (Q1, Q3)	20 (20, 40)	20 (20, 40)	0.0 (0.0, 0.62)	>0.999
Missing		3		
SPSC lag category; *n* (%)				
<20	0 (0.0)	0 (0.0)		
20–40	19 (95.0)	16 (80.0)		
>40	1 (5.0)	1 (5.0)		
Spike gamma source coherence @ 90%; *n* (%)	20 (100)	15 (75)	0.15 (0.001, 2.09)	0.177
Missing		3		
Spike gamma source coherence lag range; median (Q1, Q3)	20 (20, 20)	20 (20, 40)	0	>0.999
Missing		5		
SPGSC lag category; *n* (%)				
<20	0 (0.0)	0 (0.0)		
20–40	18 (90.0)	15 (75.0)		
>40	2 (10.0)	0 (0.0)		
Spectral power; median (Q1, Q3)	12.0 (8.4, 15.5)	9.15 (6.75, 13.0)	1.0 (−4.87, 1.57)	0.642
Mean global field power; median (Q1, Q3)	546.0 (373.75, 886.25)	376.5 (233.75, 1022.75)	17 (−237.43, 246.09)	0.932

SPSC, spike source coherence; SPGSC, spike gamma source coherence; Q1, first quartile, Q3, third quartile. *p*-value is significant at 0.05.

There was no significant difference between spikes, spike source coherence at 90%, spike source coherence lag range, spike gamma source coherence at 90% or spike gamma source coherence lag range, spectral power, or mean global field power between initial diagnosis and follow-up after treatment with vigabatrin using a logistical mixed effects model.

### EEG biomarkers for vigabatrin treatment responders and non-responders

There was a significant difference between the mean global field power of the vigabatrin non-responders at 1,017 μV^2^ (CI 356–1,403 μV^2^) and the vigabatrin responders at 276 μV^2^ (CI 222.5–508 μV^2^) with a *p*-value of 0.038.

For the vigabatrin responders, spike source coherence at 90% network size was increased in 1/8 (12.5%) case, unchanged in 5/8 (62.5%) cases, and reduced in 2/8 (25%) cases compared to the vigabatrin non-responders, which were increased in 4/9 (44%) cases, unchanged in 3/9 (33%) cases, and reduced in 2/9 (22%) cases.

For the vigabatrin responders, the spike gamma source coherence at 90% network size was increased in 5/6 (83%) cases, unchanged in 1/6 (17%) case, and reduced in 0/6 (0%) compared to the vigabatrin non-responders who were increased in 6/9 (67%) cases, unchanged in 1/9 (11%) case, and reduced in 2/9 (22%) cases. The EEG biomarkers for vigabatrin treatment responders and non-responders are presented in Table 3.

**Table 3:** Difference in EEG biomarkers between vigabatrin treatment responders and non-responders.

EEG biomarker	Follow-up vigabatrin EEG non-responders (*n* = 9)	Follow-up vigabatrin EEG responders (*n* = 11)	Effect estimate	*p*-value
Visual EEG				
Hypsarrhythmia; *n* (%)	9 (100)	0 (0.0)	—	
Spikes; *n* (%)	9 (100)	8 (72.7)	0.0 (0.0, 2.8)	0.218
Computational EEG				
Spike source coherence @ 90%; *n* (%)	9 (100)	8 (72.7)	0.3 (−0.1, 0.6)	0.285
Missing		3		
Spike source coherence lag range; median (Q1, Q3)	20 (20, 40)	30 (20, 40)	0 (−20, 20)	0.819
Missing		3		
SPSC lag category; *n* (%)			—	
<20	0 (0.0)	0 (0.0)		
20–40	9 (100.0)	7 (63.6)		
>40	0 (0.0)	1 (9.1)		
Spike gamma source coherence 90%; *n* (%)	9 (100)	6 (54.6)	0.0 (0.0, 4.6)	0.205
Missing		5		
Spike gamma source coherence lag range; median (Q1, Q3)	20 (20, 40)	20 (20, 35)	0.0 (0.0, 20.0)	>0.999
Missing		5		
SPGSC lag category; *n* (%)			—	
<20	0 (0.0)	0 (0.0)		
20–40	9 (100.0)	6 (54.6)		
>40	0 (0.0)	0 (0.0)		
Spectral power; median (Q1, Q3)	10 (7.9, 13.0)	8.3 (6.0, 12.0)	1.6 (−2.3, 6.0)	0.445
Mean global field power; median (Q1, Q3)	1,017 (356, 1,403)	276 (222.5, 508.0)	620 (20, 1,131)	0.038

SPSC, spike source coherence; SPGSC, spike gamma source coherence; Q1, first quartile; Q3, third quartile. Effect estimates were obtained using Fisher’s exact or test for proportions for the binary outcomes and Wilcoxon–Mann–Whitney *U* test for the continuous outcome. *p*-value is significant at 0.05.

There was no significant difference between the spikes, spike source coherence at 90%, spike source coherence lag range, spike gamma source coherence at 90% or spike gamma source coherence lag range, or spectral power between initial diagnosis and follow-up after treatment with vigabatrin.

### EEG biomarkers between the control arm and the intervention arm

**Figure 4:**
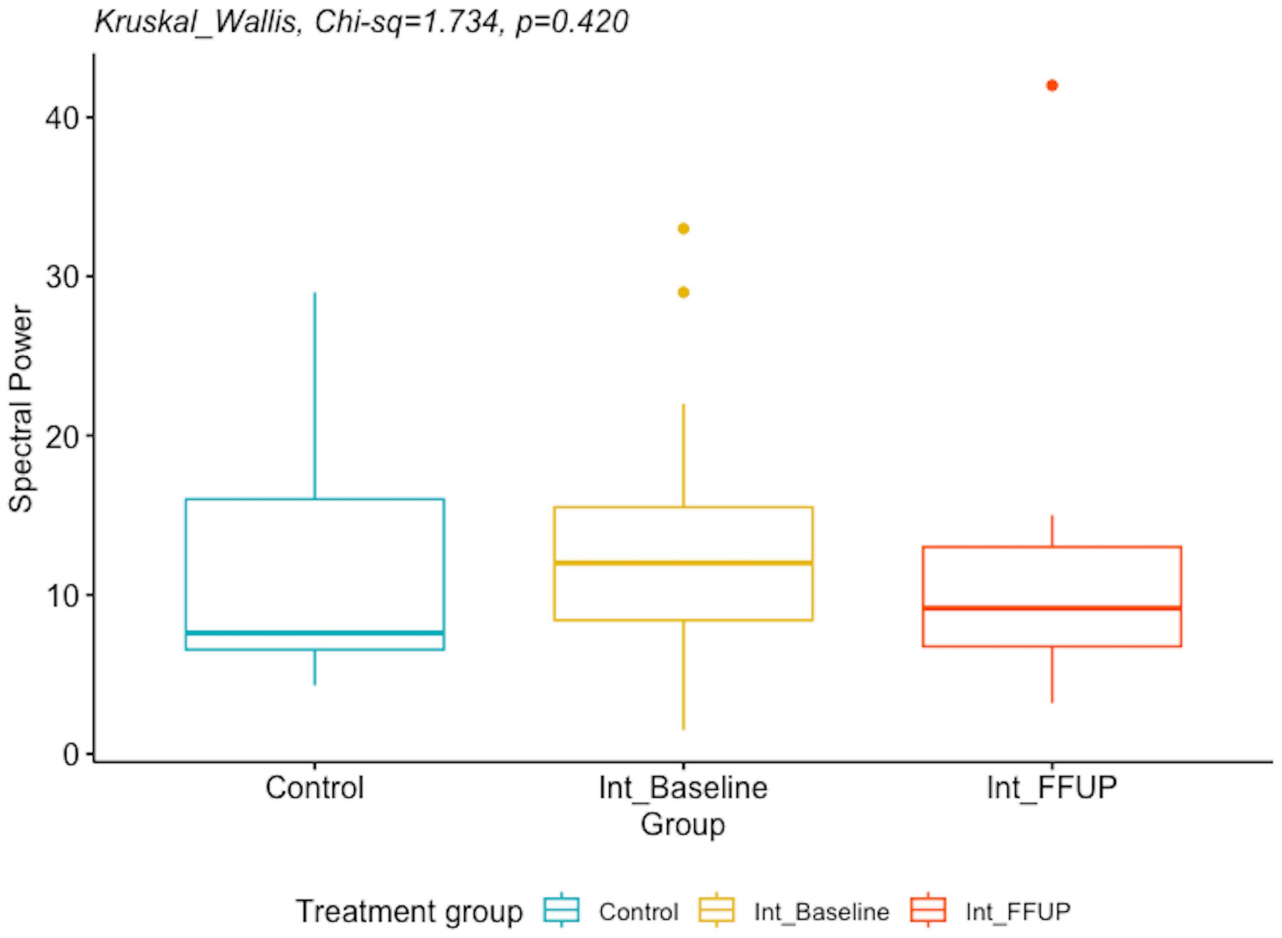
Boxplot of spectral power for controls and intervention arm at initial diagnosis and follow up.

**Figure 5:**
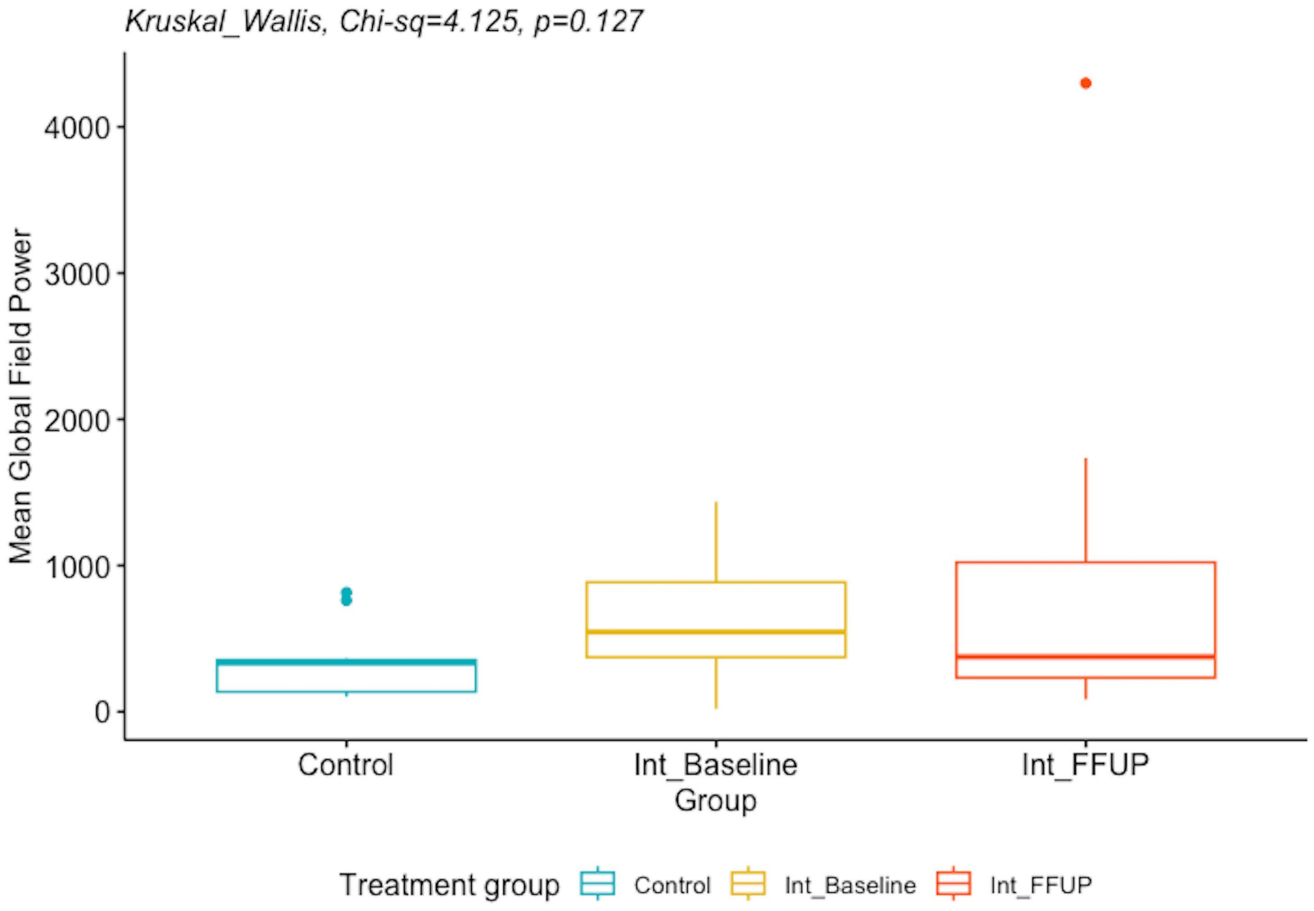
Boxplot of mean global field power for control and intervention arm at initial diagnosis and follow up.

There was no significant difference between the spectral power and mean global field power between the control and intervention groups at initial diagnosis and follow-up. The difference in EEG biomarkers between the control arm and the intervention arm at initial diagnosis and at follow-up is presented in Table 4. Boxplots of the spectral power and mean global field power for controls, as well as the intervention arm at initial diagnosis and follow-up, are shown in Figures 4, 5, respectively.

**Table 4:** Difference in EEG biomarker between control arm and intervention arm at initial diagnosis and at follow-up.

EEG Biomarker	Control (*n* = 11)	Intervention—initial diagnosis (*n* = 20)	Intervention—follow-up (*n* = 20)	Kruskal–Wallis effect estimate	*p*-value
Spectral power; median (Q1, Q3)	7.6 (6.6, 16.0)	12.0 (8.4, 15.5)	9.2 (6.8, 13.0)	1.734	0.420
Mean global field power; median (Q1, Q3)	334.0 (138.0, 356.0)	546.0 (374.0, 886.0)	376.0 (234.0, 1023.0)	4.125	0.127

Q1, first quartile; Q3, third quartile; *p*-value is significant at 0.05. Since the *p*-values from our analyses is >0.05, we conclude that there is no significant difference between the control and the intervention group at initial diagnosis and follow-up. Post-hoc analyses were therefore not performed.

## Discussion

This study correlated the interictal spike, functional connectivity networks spectral power, and MGFP in IESS at initial diagnosis with treatment response to vigabatrin using proprietary Curry software from Compumedics, Neuroscan. The difference in EEG biomarkers between the control arm and the intervention arm at initial diagnosis and at follow-up is presented in Table 4.

We found that children with IESS and hypsarrhythmia at initial diagnosis had a high degree of spike network connectivity demonstrated by >90% source coherence with a relatively short phase lag time of 20–40 ms between the 1–30 Hz and 30–70 Hz frequency ranges during sleep. Left and right hemisphere spike source coherence demonstrated strong intra and inter-hemispheric network connectivity with long-range connections from multiple brain regions.

Increased coherence may be due to synchronization of cortical activity or enhanced cortical, subcortical network connectivity (15). This finding is consistent with the results of previous studies, which showed that increased EEG coherence was associated with hysparrhythmia (15). Interictal discharges represent the summation of the paroxysmal depolarisation shifts of multiple neurons. The occurrence of interictal spikes in IESS has been associated with a global increase in connectivity without an alteration in network structure (18). Suzuki et al. (19) reported that intra- and inter-hemispheric synchronization with IESS was stronger than those with focal epilepsy after perinatal arterial ischemic stroke. The corpus callosum and other interhemispheric commissures and fiber tracts are the likely conduits for focal spike network transmission to the contralateral hemisphere. In this study, we chose to study gamma fast oscillations, which predominate over Ripple oscillations in IESS (20). Spike fast oscillations are considered pathological compared to physiological fast oscillations, which are not associated with spikes (23). Spike fast oscillations (40–200 Hz) have been shown to correspond to the severity of epileptic encephalopathy in children with tuberous sclerosis complex (22).

Electrographic resolution of hypsarrhythmia with vigabatrin occurred in 11/20 (55%) cases, which is comparable to previous studies (6). After treatment with vigabatrin, we did not find a significant reduction in spectral power. However, the MGFP at follow-up was significantly lower in the vigabatrin responders compared to non-responders.

There is little research on the dynamics of MGFP and its response to vigabatrin administration. MGFP is a measure of the cumulative strength of all electrodes in a given field across time (24). The significant decrease in MGFP observed in vigabatrin responders relative to non-responders could suggest that the increased GABAergic inhibition mediated by vigabatrin effectively reduces abnormal activity and connectivity. This reduction in aberrant neuronal activity could potentially be reflected and observed in the overall strength of the EEG map.

In contrast, although there was a decreasing trend when comparing MGFP from initial diagnosis to follow-up, this finding was not statistically significant. Larger studies are required to ascertain whether these differences reflect pre-existing baseline differences between the two groups or serve as indicators of differential responses to treatment.

There was a decreasing trend in spectral power findings from initial diagnosis to follow-up in the current study, but the findings did not yield any statistically significant results. This is consistent with a previous finding by Kim et al. (25) that found no statistical significance in spectral power between vigabatrin responders and non-responders.

A high degree of spike network connectivity remained, with no significant difference before or after treatment with vigabatrin in the presence of spikes, spike source coherence at 90%, spike source coherence lag range, spike gamma source coherence at 90%, or spike gamma source coherence lag range.

Changes in source coherence network size in the 1–30 Hz and gamma bandwidth suggest that functional connectivity is a dynamic process that may increase or decrease with vigabatrin treatment. No change or a decrease in source coherence network size at 1–30 Hz was more likely, while spike gamma source coherence network size tended to increase with vigabatrin.

Spike source coherence network size increased less for vigabatrin responders than for vigabatrin non-responders between 1 and 30 Hz, while spike gamma source coherence network size increased more for vigabatrin non-responders.

This suggests that the response of spike source coherence connectivity in sleep to vigabatrin alone may be lower than the response of connectivity using cross-correlation coherence of epochs of awake background brain wave activity after adrenocorticotropic hormone (ACTH) or vigabatrin treatment as described by Shrey et al. (17) They found that responders showed decreased connectivity, while non-responders exhibited minimally decreased or increased connectivity after treatment.

Given that measures of network coherence have frequently been found elevated in IESS relative to controls, further studies with higher numbers and longer follow-up intervals are important to ascertain whether differences exist in these parameters post-treatment with vigabatrin, whether responses in these parameters are simply slower to manifest, or whether these changes reflect an element of the underlying pathology that is not modified by vigabatrin.

Increased spike network connectivity, likely due to synchronization of cortical activity or enhanced cortical-subcortical network connectivity in IESS, may represent hyperexcitable neuronal circuits that increase the risk for seizures and epileptogenesis during this critical period of brain development. IESS occurs during infancy, a time when neuronal glutaminergic excitation predominates to promote synaptogenesis in cortical and limbic neuronal networks, resulting in increased synaptic and spine density, as well as elevated excitatory ion channels and transporters, alongside a relative reduction in GABAergic inhibition (2). Vigabatrin, a GABA transaminase inhibitor, works by increasing GABAergic inhibition in interneurons and reducing neuronal excitation, potentially leading to the resolution of hypsarrhythmia.

This study reinforces the potential of computation EEG biomarkers to complement visual biomarkers in the diagnosis and management of IESS. Specifically, this study identified post-treatment differences in MGFP between vigabatrin responders and non-responders, raising the possibility that this biomarker—alone or in combination with others—could play a role in identifying responders. Further studies involving a larger number of cases will be required to determine the relationship between MGFP elevation and response to vigabatrin, as well as its practical clinical utility.

### Limitations

This study was a retrospective analysis with a small sample size. A key concern when evaluating connectivity is the effect of volume conduction; therefore, we removed zero-lag time cross-correlation to mitigate this issue (17). We also used low-density EEG with 21 channels, which is standard for clinical EEG, instead of high-density research EEG recordings. We also used a standard pediatric MRI head model instead of individualized MRI head models. The EEG sampling rate also limited the recording of fast ripples.

## Conclusion

These findings support the hypothesis that IESS is a disorder characterized by increased neuronal activity and network connectivity. MGFP is a potential computational biomarker for assessing response to vigabatrin. Future studies should explore whether coherence in IESS is related to its underlying etiology (15). Additionally, larger prospective studies are needed to assess the clinical significance of computational biomarkers in measuring treatment response in IESS.

## Data Availability

The raw data supporting the conclusions of this article will be made available by the authors, without undue reservation.
